# A machine learning method for predicting telescope cycle time applied to the Cerro Murphy Observatory

**DOI:** 10.1007/s10686-024-09970-8

**Published:** 2024-11-28

**Authors:** Mirosław Kicia, Mikołaj Kałuszyński, Marek Górski, Rolf Chini, Grzegorz Pietrzyński

**Affiliations:** 1 Nicolaus Copernicus Astronomical Center, Polish Academy of Sciences, Bartycka 18, Warsaw, 00-716 Poland; 2 Ruhr University Bochum, Faculty of Physics and Astronomy, Astronomical Institute (AIRUB), 44780 Bochum, Germany; 3 Universidad Católica del Norte, Instituto de Astronomía, Avenida Angamos, 0610 Antofagasta, Chile

**Keywords:** Machine learning, Software, Instrumental astronomy, Observation scheduling

## Abstract

Telescope cycle time estimation is one of the basic issues of observational astronomy. There are not many tools that help to calulate the cycle time for multiple telescopes with multiple instruments. This work presents a new tool for determing the observation time; it was applied at the Cerro Murphy Observatory (OCM) but can be used at any other observatory. The Machine Learning (ML) method was implied, resulting in a fully automatic software module that works without any user intervention. We propose a polynomial multiple regression method and demonstrate all steps to build a reliable ML model like data collecting, data cleaning, model training and error evaluation in relation to the implementation in the observatory software. The method was designed to work for different telescopes with several instruments. Accuracy analysis and the assessment of model errors were based on real data from telescopes, proving the usefulness of the presented method. Error evaluation shows that for 84.2 % of nights, the prediction error in operation time prediction does not exceed 2 %. Converted into a 10-hour observation night, 2 % corresponds to an error of no more than 12 minutes. The described model is already working at the OCM and optimizes the efficiency of the observations.

## Introduction

The telescope cycle time estimation (prediction) is one of astronomy’s instrumental issues, which accompanies every astronomer in their daily work, primarily for observation scheduling. Accurate time scheduling is becoming more important in today’s increasingly automated, fully or partial robotic observatories. Some observatories dissolve into partially or fully commercial ones so telescope up-time becomes directly related to revenue. Telescope cycle-time prediction is a necessary legal factor for proper and accurate observation scheduling. There are many situations in which timely observations are crucial, e.g. observations of eclipsing binaries, pulsating stars or other on-time astronomical events [1]. Therefore, it is crucial to place an observation accurately in order not to miss a specific object phase or to miss the event at all. The time of an observation determines the accuracy and the usefulness of the data and an efficient scheduling increases the scientific efficiency. Several articles address observation scheduling for observatories such as ALMA [2–4], HST [5, 6], and others without a specified designated observatory [7–9], but none explore the telescope cycle time calculation itself. Most of the studies focus on higher-level scheduling issues, emphasizing flexible, accurate, and efficient scheduling. Garcia-Piquer et al. [10] describe the process of calculating overhead time, which is later used in observation scheduling. The method relies on time values that are likely measured manually and leads to the establishment of constant overhead time coefficients.

Since the aforementioned sources contain limited information about telescope cycle time estimation the problem was compared to lead time calculations in the production area. There are many sources in manufacturing that describe the time prediction problem, from general theories to specific methods for specific tasks. Chung and Huang [11] proposed four categories for production cycle time estimation (i) simulation, (ii) statistical analysis method, (iii) analytical method and (iv) hybrid method. Chen  [12] extended the number of methods to six (i) statistical, (ii) production simulation (PS), (iii) artificial neural networks, (iv) case-based reasoning (CBR), (v) fuzzy modeling, and (vi) hybrid approaches.

Lee and Gao [13] prepared a wide overview of manufacturing cycle time calculation methods within the above classifications, and based their hybrid model on some ML elements: c-means clustering with genetic algorithm and backpropagation network predictor.

A statistical analysis method used by Raddon and Grigsb [14] and Yang [15] proposed methods based on a regression model. Backus et al. [16] proposed a statistical method based on data mining and described features and characteristics of the cycle-time problem. This work also uses combined ML methods: clustering, k-nearest neighbours and regression trees.

The use of neural networks increases in recent publications on batch time calculation. Sha and Hsu [17], Chien et al. [18], and Gazder & Ratrout [19] described methods based solely on neural networks with an admixture of other methods.

The main discrepancy between the above sources and our task is that in most cases the time is estimated for the entire batch and does not distinguish between subsequent stages. The above works had useful implementations of ML methods elements, but their main trend was deeply embedded in production which did not allow to transfer these methods directly to our task.

## Motivation

The aim of this work was to develop a reliable software for the OCM which is the part of The Araucaria Project [20]. This facility is currently undergoing a major upgrade including four new telescopes, infrastructure and software. The new OCM software is prepared from scratch in Python using modern frameworks and computation methods. The OCM has already four telescopes operational while a new 2.5 m telescope is under construction (see Table 1). The multitude of telescopes and instruments required a new method to calculate the telescope cycle time.

**Table 1:** OCM telescopes

Name	Aperture	Type	Focal Length	Mount Type	Instruments^a^
ZB08	80 cm	Ritchey-Chrétien	548 cm	alt-az	CCD
JK15	150 cm	Ritchey-Chrétien	900 cm	alt-az	CCD, S
WK06	60 cm	Ritchey-Chrétien	420 cm	equatorial	CCD
IRIS	80 cm	Cassegrain	500 cm	alt-az	IR
WG25^b^	250 cm	Ritchey-Chrétien	1750 cm	alt-az	CCD, IR, S

Note: Multitude of telescopes and instruments at the OCM

^a^ CCD - CCD optical camera, IR - infrared camera, S - spectrograph

^b^ Telescope under construction

Initially, we considered different approaches to solve the problem, including analytical, simulation or ML methods. A first analysis showed that the analytical approach implied some unacceptable drawbacks. Given the large number of telescopes and instruments such an approach would involve measuring all time parameters, which is inefficient and time-consuming. Our experiments have shown that for a larger number of parameters the analytical method is more accurate, however, increases the workload. Therefore, this method does not meet the requirement of being maintenance-free. We also found no arguments to use the simulation method, as simulation methods are typically employed in scientific contexts where experimental solutions are either impractical or infeasible. In summary, the analysis of ML methods seemed more promising than all other methods.

Every telescope has a different construction resulting in different parameters and speed; the same is true for the instruments, like download time, readout speed and preparation time. Even measuring all these values one cannot be sure that the telescope cycle-time will be predicted with sufficient accuracy. Because every software task generates some time lags in some random moments which can not be determined efficiently, the computer control unit of the telescope may experience deceleration in its performance. However, with certain hardware upgrades the processing speed can be increased. Consequently, the time delays associated with the computer’s tasks will vary over time.

**Fig. 1:**
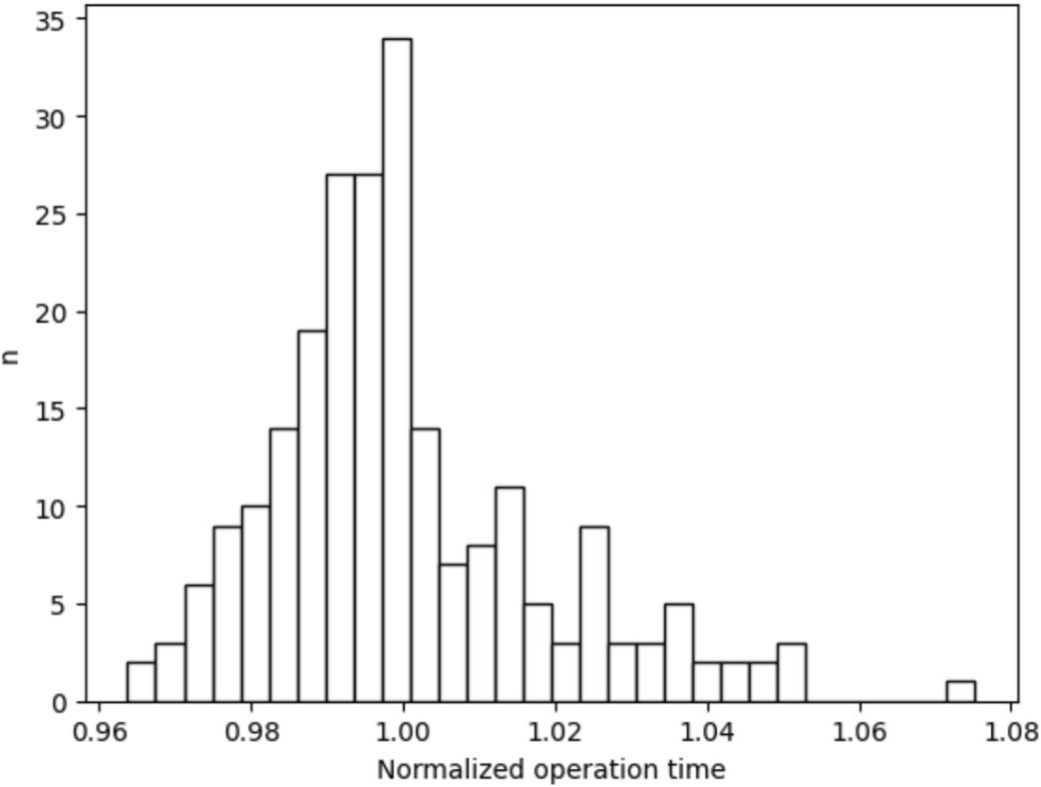
Histogram of normalized (by mean OP time) WK06 telescope OP cycle time with same observation parameters. The standard deviation is $SD_{s}$ = 0.0182 and median $Me_{s}$ = 0.997

In our case, the calculation of the telescope time was based on the calculation of the operation (OP, see Section 3.1) time. To determine the maximum accuracy of the OP time prediction, we measured the OP time for the same OP parameters and normalized them by the average OP time as shown in Fig. 1. This figure shows that the same task for the same telescope takes different OP time values.

All telescopes parameters like mount speed or different moving strategies can be changed during the telescope use, i.e. all time parameters have to be newly measured. The same situation holds for the instruments parameters, because they can be changed too. Even the instrument itself can be replaced with another one requiring a different set of time parameters.

The time calculation becomes even more complicated because some sub-operations are performed simultaneously while others are not. Some sub-operations, e.g. filter change, have different time lengths because of their specification. Obviously, depending on the current filter slot, the so-called length of time can vary by a factor of tens to reach the next filter position. In OPs where the exposure time is very short but the number of filter changes is high, a significant fluctuation in OP time length may occur.

The ML methods solve telescope cycle-time calculation problem with several advantages: i) Access to data is quite easy. ii) The database containing the OP time and the parameters, increases, allowing to retrain the model with greater accuracy. iii) ML methods do not demand any additional measurements of time and can find these values automatically. iv) The ML methods can learn from historical data and react to changes without any outside interference [21]. v) Changing the time parameters will not affect the results because the model will learn on the fly.

## Model asumptions

### Operation (OP) time

The basic unit of calculating telescope time estimation is a single OP time *t*. In the OCM control system the OP can be related to a single observational block (OB). A set of OBs constitutes a night observation plan. The sum of OP times *t* equals the total duration of the observing night. The OB consist of an OP type (i.e. OBJECT, DARK, SKYFLAT, ZERO etc.) and arguments. The arguments depend on the OP type; the most important ones for the time estimation were sequence *seq*, coordinates *coo* and *dither* option. The *seq* contains information about exposures, filters and integration time, *coo* are right ascension and declination of the target and *dither* decribes the offsets for differential measurements. Here is an OB block example:
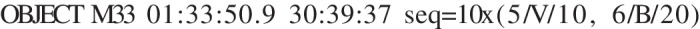


This results in executing an OP type OBJECT on the target M33 with the coordinates 01:33:50.9 30:39:37 and a *seq* of 10 times 5 exposures in the *V* filter with 10 seconds exposure time each and 6 exposures in the *B* filter with 20 seconds exposure. The OP type OBJECT has per default the dithering option “off” so there is no dithering in this case. Underneath, every OP is decomposed into a number of sub-operations such as mount, dome slew, filter change, exposure execute and others. Some sub-operations may run simultaneously, but a strictly sequential mode can be enforced for easier debugging and testing.

### Estimation algorithm

The variable *t* is continuous which reduces the number of possible ML models but still leaves a wide selection possibility. We selected regression, neural networks and all “regressor” kind models and made the following assumptions. The model had to have a sufficient level of complexity to meet the task, but at the same time it should be easily manageable. We tried to achieve a simple solution with satisfactory results like good accuracy and high model interpretability. Therefore, we did not consider the “black box” [21] models to had more control on parameters and on inner steps values.

Another criterion involved how the model and data were stored. The first type generates only parameters that have to be stored while the second case stores the model itself as a parametric model in form of an object serialization. It is easier to manage parameters than object serialization models which are difficult to store and are not as transparent as parameters.

Hence, the choice was narrowed down to regression models. The Polynomial Regression (PR) was the model that met our conditions best. The PR is variant of Multiple Linear Regression with polynomial features which are increasing the model capacity; the latter one describes the ability to solve more complicated tasks. It has to be as close as possible to the complicity and the variation of the task. If this parameter is badly selected the model can overfit or underfit the result [21].

PR model equation:

1 $$\begin{aligned} y_{\beta }(x) = \beta _0 + \beta _1 \cdot x_1 + \beta _2 \cdot x_2^2 + \beta _3 \cdot x_1 \cdot x_4 + \dots \end{aligned}$$

where $\beta _0$ is the intercept and $\beta _i$ for i = {1, 2, ..., n} are coefficients.

The PR model is a supervised algorithm where we had to manually select features, unlike neural networks where the function selects features automatically. The construction of the PR equation was done from experience and from our analysis.

The PR equation in our case is

2 $$\begin{aligned} y = \beta _{dome} \cdot D + \beta _{mount} \cdot M + \beta _{expos} \cdot E + \beta _{filter} \cdot F + \beta _{read} \cdot R \cdot N + \beta _{dither} \cdot T \cdot N + \beta _0 \end{aligned}$$

where, *D* is the dome distance passed in OP in $\deg$, *M* is the mount distance passed in OP in $\deg$, *E* is a sum of integration times for the whole OP, *F* is number of filter changes, *N* is number of exposures and *T* is the dithering option on / off, where on is equal 1 and off is equal 0. The readout mode factor *R* is calculated from:

3 $$\begin{aligned} R = \frac{1}{r} \end{aligned}$$

where, *r* is readout mode in MHz. The simple features are *D*, *M*, *E*, *F* and polynomial features are $R \cdot N$ and $T \cdot N$.

Each term in (2) is responsible for some time-consuming task that we are able to calculate from the parameters entered into the OB or from parameters of the telescope. For example, *D* was calculated from initial dome position and *coo* while *M* were estimated from the initial mount position and also *coo*. The quantities *E*, *F*, *N* and *T* were calculated from the *seq* while *R* was taken from telescope parameters. The polynomial terms $R \cdot N$ and $T \cdot N$ in (2) change with the numbers of exposure because dithering and readout time are strictly connected to *N*.

To calculate the coefficients $\beta$ in (2) we need to train the model (see Section 3.4). After the training the coefficients took values of particular time ingredients, e.g. dome speed $\beta _{dome}$ in second/$\deg$, the changing time of a filter $\beta _{filter}$ in seconds and a single dithering movement $\beta _{dither}$ also in seconds. The coefficient values were very usefully to compared to real values.

This form of (2) is appropriate for most instruments and mounts types. If additional time-consuming tasks arise, the equation can be extended by adding more terms. However, it is important to always strive for maintaining simplicity. The coefficients and intercept provided some generalisation space; minor missing time ingredients will automatically be moved by the training to the intercept $\beta _0$.

If some events are not existing in our system it is better to remove them from  (2) to prevent too many degrees of freedom for the training. Likewise this could have an impact on underfitting or overfitting the results.

Another important issue is the assessment of selected features in terms of their correlation. Because the PR model is linear, high correlations between features are not allowed and can affect the results. Table 2 shows correlations between features; there a higher correlation value occurs between dome distance and mount distance. This high correlation value is obvious because usually if the dome has a long way to go the mount has it too. However, moving the telescope only in altitude the dome distance is not changing at all. Because both factors are correlated, but there is no causal relationship, it was decided not to remove one of these two features. High correlation should be regarded solely as an advisory factor but not as a determining one.

**Table 2:** Zb08 telescope OP type OBJECT feature corellation

	D	M	E	F	$R \cdot N$	$T \cdot N$
D	1.000000					
M	0.922331	1.000000				
E	0.266929	0.269947	1.000000			
F	0.173578	0.178091	0.005855	1.000000		
$R \cdot N$	0.007963	0.017147	$-0.024346$	$-0.054667$	1.000000	
$T \cdot N$	0.036573	0.054037	$-0.024463$	$-0.125984$	0.090050	1.000000

**Table 3:** Example of the OP type OBJECT cleaned data for the Zb08 telescope

Cycle time	D	M	E	F	$R \cdot N$	$T \cdot N$
225.314	105.666	62.818	48.0	3	16.0	0
274.211	135.788	82.720	46.0	3	22.0	0
351.767	39.881	33.941	46.0	3	22.0	22
241.323	49.976	41.583	124.0	2	11.0	0
310.203	80.521	67.601	46.0	3	22.0	22

Cycle time is target variable and rest six collumns are the features

Note: OP id and time stamp not shown

### Data collecting and cleaning

First, data stamps from the beginning and the end of the OP were collected; this data is called raw data. The data stamp contains all possible information that is available at this stage. The OP beginning data stamp contains the dome azimuth position, the mount azimuth and altitude position, identification, OB identification, OP type, parameters like *seq* or *dither*, UTC time stamp, instrument readout mode and active filter position. The OP end data stamp has the same records (some with different values) plus an OP status flag that describes the OP status if it was successful or skipped. The process of collecting raw data, unlike data cleaning, does not change due to the model changes.

The data cleaning process, which uses raw data, is rejecting wrong data and is preparing a data set to train the model. This stage is strictly connected to PR model shape; changing the model means changing the data cleaner too. The data cleaner is calculating dome and mount distance passed in deg, OP time in seconds, number of filter changes, number of dithered expositions, and the readout mode factor multiplied by the number of exposures (Table 3). The module differentiates the data records from the begin and the end of an OP. If the raw data have a begin data stamp but miss an end data stamp the record is rejected; it could mean that the OP was interrupted. The data cleaning module also omits skipped OPs.

To be more precise to specific OP types the cleaned data is split according to the OP type and is saved as separate files. The OP type OBJECT which is a celestial object observation is most important for the accuracy of the time calculation task because they comprise the largest number in a night observation plan. The data separation allowed us to not contaminate the OP type OBJECT data with other OP types.

For example the OP type DOMEFLAT has different time lags and delays than other OPs which could impact the coefficients of the trained model. The OP type OBJECT data will impact the SKYFLAT coefficients because only few OPs compare the OBJECT OPs number.

The data for different instruments are also split. Each telescope has a data set for each instrument and these contain data for each OP separately. This approach is a safe approach. Theoretically, keeping all data in one file should be sufficient, but dividing the data into instruments and OPs and train them separately will increase accuracy.

### Model training

The PR model (2) was built in python Scikit-learn, ML python library [22]. This model uses mean squared error as a loss function. Due to the expected large amount of data, which in itself has an anti-overfitting effect [21], regularization was not used.

The data used for training was split into the training set (70%) and the test set (30%). Because of a data split by OP type (see Section 3.3) we did the training for every OP type and for every instrument separately. Any ML model needs data to train the model and to predict the results. To achieve satisfactory results a minimum number of data records is required. This minimum number is not obvious and we started with 10 records; below this number no *t* is predicted. It is recommended to perform 10 to 20 observing blocks in the beginning. After this short introduction the system starts to provide results for *t*.

The system of coefficients will only work properly if the model is trained with data over a wide range. Using, e.g., only a readout mode of 1 MHz, $\beta _{read}$ will vanish but the accuracy of time prediction will stay constant. If, however, in the future a value of 3 Mhz will be used, the first predictions will not be very precise. After some model trainings the situation will be fixed automatically. Therefore, to prepare for all configurations, it is recommended to perform the number of OPs with all possible combinations of parameters during the initial use of the telescope.

### Software implementation

As mentioned in Section 2 the goal is to create an autonomous method to calculate the cycle-time without any human interference. The module is split into four classes:

- data collector
- data cleaner
- trainer
- calculator

The data collector is implemented inside a plan runner module. The data stamps are taken during the running night plan. Data cleaner and trainer are run when the observer leaves the user interface (GUI). These two activities may take some time and leaving the GUI is the most save moment to execute them. The cleaned and trained data are placed in some network location which can be accessed by the time calculator. If some client (GUI, other software, service) needs a time prediction, the calculator modul will access trained coefficients and forward the result to the client.

## Results

### Time prediction

**Fig. 2:**
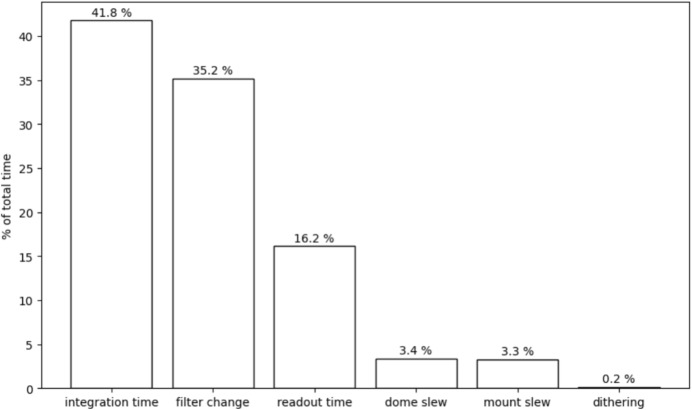
Sample chart of the ZB08 telescope. The total time ratio of time-consuming tasks is shown for a model trained on 3074 observations

We have not measured the time of individual sub-operations, but used trained coefficient values and parameters to construct a summary chart Fig. 2 showing the ratio of the total time of time-consuming tasks, as represented in (2). This chart is helpful to evaluate the telescope efficiency and allows for more program or hardware improvements. On the other hand, it strongly depends on observation strategies, as well as mount and instrument parameters. Many short exposures of bright objects versus long exposures of faint objects will yield different charts for the same telescope.

Table 4 shows an example of trained model coefficients. Compared to (2) the meaning of the coefficients becomes more clear now. The coeficients $\beta _{dome}$ and $\beta _{mount}$ denote the speed of mount and dome, respectively. $\beta _{filter}$ is the average time of one filter change, $\beta _{read}$ is actually the time taken for one exposure (without integration time), and $\beta _{dither}$ is time taken for one exposure dithering.

To obtain an OP time prediction the values *D*, *M*, *E*, *F*, *R*, *N*, *T* values (see (2)) are required. *D* and *M* were calculated from OB coordinates and from the current telescope and mount positions. *E*, *F*, *R*, and *N* are taken from sequence *seq*. Taking an OB example from Section 3.1, Equation (2) will attain the form:

4 $$\begin{aligned} y = 0.12 \cdot 10 + 0.24 \cdot 15 + 1.02 \cdot 1700 + 4.87 \cdot 20 + 4.84 \cdot 1 * 110 + 5.15 \cdot 0 \cdot 110 + 3.34 \end{aligned}$$

5 $$\begin{aligned} y = 2372 \sec = 39.53 \min \end{aligned}$$

**Table 4:** Example of coeficients values trained on OP type OBJECT Zb08 telescope data

$\beta _{dome}$	$\beta _{mount}$	$\beta _{expos}$	$\beta _{filter}$	$\beta _{read}$	$\beta _{dither}$	$\beta _0$
0.12	0.24	1.02	4.87	4.84	5.15	3.34

### Error evaluation

To evaluate the model and model prediction errors, calculations for 3074 and 7339 observations were performed. The main accuracy indicator for PR was the R-Squared (later $R^2$) error. For 3074 observations $R^2_{3074}$ = 0.9837 and for 7339 observations $R^2_{7339}$ = 0.9980, so the difference is negligible. This gives the impression that the models have the same accuracy which does, however, not represent the whole truth.

Prediction error for observation will attain the form:

6 $$\begin{aligned} e = \frac{MCT - PCT}{MCT} \cdot 100 \% \end{aligned}$$

where, *e* is prediction error % for observation, *MCT* is measured cycle time and *PCT* is predicted cycle time.

**Fig. 3:**
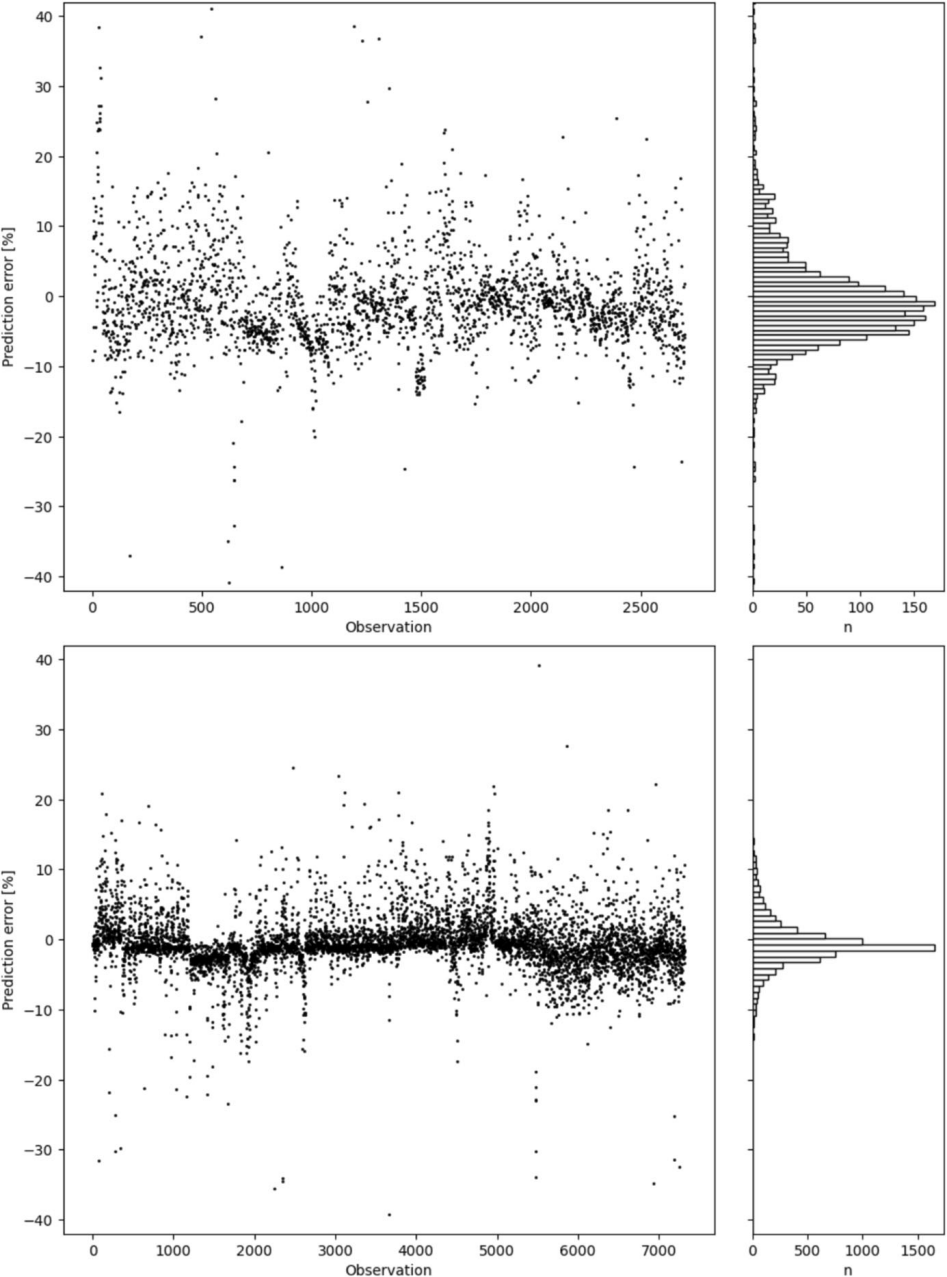
Prediction error for all observations for the Zb08 telescope of the two samples; 3074 (up) and 7339 (down)

Figure 3 shows an observations prediction error and their histograms for the Zb08 telescope. Table 5 shows the error evaluation for the Zb08 telescope. For the model trained on 3074 observations the prediction error standard deviation is *SD* = 8.41% and the prediction error median is *Me* = -1.54%. For the model trained on 7339 observations the corresponding values are 3.94% and -0.92%, respectively. Both *SD* and *Me* values were calculated after applying a 3-sigma clipping to the results. Table 5 also shows the percentage of observations with prediction errors below the thresholds of 2%, 1%, and 0.5%. Assuming a 10-hour observation night, these thresholds correspond to prediction errors of 12 min, 6 min, and 3 min for the entire night, respectively.

**Table 5:** Error evaluation for the Zb08 telescope

				Percent of observations below error e		
Observations	$R^2$	SD	Me	$e < 2$ %	$e < 1$ %	$e < 0.5$ %
3074	0.9837	8.41 %	$-1.54 \%$	76.1 %	69.8 %	66.6 %
7339	0.9980	3.94 %	$-0.92 \%$	84.2 %	77.8 %	73.2 %

## Discussion and conclusion

So far, the telescope cycle time prediction has not been widely analyzed. While the topic initially appears straightforward and elementary, it becomes considerably complex upon engaging in detailed calculations.

Our main goal was the simplicity of the solution while ensuring accuracy. The model is based on one (2) which is simple, interpretable, and it is avoiding the “black box” models problem. The features used to build the model are usefull to evaluate and to compare with real telescope time parameters.

As mentioned in Section 1, compared to solutions, where cycle time is calculated for the whole batch production process, our way of modeling is more specific, because we predict the OP time of one machine, i.e. a telescope. The mentioned articles do not divide the analysis into individual machine operations, but try to predict the time of entire batches, which disqualified them from being used for our task.

The analysis of the results in the Section 4.2 shows that the $R^2$ value for both samples (3074 and 7339) are very high, which means that the model fits the data well in both cases. Further analyses showed that a larger number of data allows for greater accuracy in time prediction. The prediction error (see (6)) standard deviation decreased from 8.41% to 3.94% while the prediction error median decreased from -1.54% to -0.92%. In both cases the median is negative which indicates that the time prediction is a bit shorter than the real value. Both results also indicate that with more training observations the variable is approaching the target of median equal to zero and reducing its dispersion as seen on Fig. 3.

The most valuable factors describing the model’s performance are the percentages of observations for which the prediction error did not exceed the thresholds of 2 %, 1 %, and 0.5 %. These thresholds can be understood as the prediction error of a single OP but also the time prediction error of the entire observation night. Assuming a 10-hour observation night threshold of 2 % can be converted to 12 min error. Table 5 shows that, for the model trained on 7339 observations, 84.2 % of the predictions have prediction error below 2 %, 77.8 % of the predictions have prediction error below 1 % and 73.2 % of the predictions have prediction error below 0.5 %. Converting this to the time of the entire observation night, we can conclude that 84.2 % of the observation nights will have a prediction error of 12 minutes or less, 77.8 % of the observation nights will have a prediction error of 6 minutes or less, and 73.2 % of the observation nights will have a prediction error of 3 minutes or less.

The method is currently working at the OCM with very satisfactory results. Since there are only two observers and four telescopes (in the future even five), the reduction in workload caused by the automatic time calculation for each telescope and instrument is noticeable. This method can be used for most of the observatories with any number of telescopes and multiple instruments. Using this method is a step towards creating an autonomous observatory. It might also be an inspiration to calculate operation times in other areas.

The control software at the OCM is constantly being developed. For this reason, some historical observations data stamps are far from expected, but we decided to not remove them. The data has not been sanitized in any way to demonstrate the system’s fault tolerance. The level of complexity included in the model is sufficient for the task.

## Data Availability

The datasets and code utilized in this study are available on demand from the corresponding author.

## Software

Software used in this work: Scikit-learn [22], NumPy [23], Matplotlib [24], Seaborn [25] and Pandas [26].
